# Effects of Fully Open-Air [CO_2_] Elevation on Leaf Photosynthesis and Ultrastructure of *Isatis indigotica* Fort

**DOI:** 10.1371/journal.pone.0074600

**Published:** 2013-09-18

**Authors:** Xingyu Hao, Ping Li, Yongxiang Feng, Xue Han, Ji Gao, Erda Lin, Yuanhuai Han

**Affiliations:** 1 College of Agronomy, Shanxi Agricultural University, Taigu, China; 2 Key Laboratory of Ministry of Agriculture on Agro-environment and Climate Change, Institute of Environment and Sustainable Development in Agriculture (IEDA), Chinese Academy of Agricultural Sciences, Beijing, China; 3 Key Laboratory of Crop Gene Resources and Germplasm Enhancement on Loess Plateau, Ministry of Agriculture, Institute of Crop Genetic Resources, Shanxi Academy of Agricultural Sciences, Taiyuan, China; 4 College of Agronomy, Heilongjiang Bayi Agricultural University, Daqing, China; University of North Dakota, United States of America

## Abstract

Traditional Chinese medicine relies heavily on herbs, yet there is no information on how these herb plants would respond to climate change. In order to gain insight into such response, we studied the effect of elevated [CO_2_] on *Isatis indigotica* Fort, one of the most popular Chinese herb plants. The changes in leaf photosynthesis, chlorophyll fluorescence, leaf ultrastructure and biomass yield in response to elevated [CO_2_] (550±19 µmol mol^–1^) were determined at the Free-Air Carbon dioxide Enrichment (FACE) experimental facility in North China. Photosynthetic ability of *I. indigotica* was improved under elevated [CO_2_]. Elevated [CO_2_] increased net photosynthetic rate (*P*_N_), water use efficiency (WUE) and maximum rate of electron transport (*J*_max_) of upper most fully-expended leaves, but not stomatal conductance (g_s_), transpiration ratio (*T*r) and maximum velocity of carboxylation (*V*_c,max_). Elevated [CO_2_] significantly increased leaf intrinsic efficiency of PSII (*Fv’/Fm’*) and quantum yield of PSII(*ΦPS* ***II***), but decreased leaf non-photochemical quenching (*NPQ*), and did not affect leaf proportion of open PSII reaction centers (*qP*) and maximum quantum efficiency of PSII (*Fv/Fm*). The structural chloroplast membrane, grana layer and stroma thylakoid membranes were intact under elevated [CO_2_], though more starch grains were accumulated within the chloroplasts than that of under ambient [CO_2_]. While the yield of *I. indigotica* was higher due to the improved photosynthesis under elevated [CO_2_], the content of adenosine, one of the functional ingredients in indigowoad root was not affected.

## Introduction

Global atmospheric CO_2_ concentration ([CO_2_]) is predicted to reach 550 µmol mol^–1^ by the middle of this century [1]. The increase in [CO_2_] may improve the photosynthetic efficiency of plants, thereby increasing the supply of photoassimilates, dry mass and yield [2]–[4]. Higher [CO_2_] increases the carboxylation rate of Rubisco but inhibits the oxygenation of Ribulose-1, 5-bisphosphate (RubP) [3]. These [CO_2_] effects vary with crops, cultivars and plant development stages.

Chinese medicinal herbs are among the oldest alternative and complementary medicines. Their ever-increasing use indicates public interest in such medicines and their important roles. Chinese herbal medicines constitute multi-billion-dollar industries worldwide and 1500 herbals are sold as dietary supplements or ethnic traditional medicine. It is expected that there would be a greater boost in the use of Chinese herbal medicine [5]. Consumers spending on herbal products in the United States was estimated to be more than $5 billion per year, mainly from self-prescription of over-the-counter products [6]. The research on traditional Chinese medicine has been taken more and more seriously [7], [8].

*Isatis indigotica* Fort, grown and used in most regions of China, belongs to the *Brassicaceae* family. The dried root and leaf of *I. indigotica*, or indigowoad root and indigowoad leaf, can be used as medicine according to Chinese Pharmacopeia [9]. *I. indigotica* is biennial, with the leaf and root from the first but not the second year being used as medicine. Seeds are harvested in the second year. *I. indigotica* has multiple pharmacological properties such as anti-viral, anti-cancer, anti-bacterial and immune enhancement [10], [11]. It can reduce fever, detoxify and benefit the pharynx [12]. Adenosine is one of the main medical ingredients of indigowoad root [13], [14], and is an indicator of the quality of indigowoad root [14]. Adenosine can be used as cardioprotective and therapeutic agent for chronic heart failure [13], [14]. It also shows anti-inflammatory efficacy [13], [15]. The most popular medicine form of the herb is indigowoad root infusion, which is used for treating flu. The current annual consumption of indigowoad root was about 2 million kg in China [16].

Understanding how elevated [CO_2_] affects herbal medicine plants is important to generate information for farmers and industries on how to respond strategically to climate change. Yet, there is no research on the effect of [CO_2_] on the leaf photosynthetic physiology and growth in herbal medicine plants under open-air conditions. The response of *Arabidopsis thaliana* and Chinese Cabbage, both belong to the *Brassicaceae* family, have been investigated using FACE and enclosure studies. Elevated [CO_2_] significantly increased leaf photosynthetic rate, but reduced stomatal conductance and transpiration rate in *Arabidopsis thaliana* and Chinese Cabbage [17], [18].

The maximum electron transport rate (ETRmax) of light reactions of photosynthesis, one of the chlorophyll fluorescence parameters to evaluate the changes of leaf photosynthesis, was reported to increase under elevated [CO_2_] [19]. Elevated [CO_2_] could also change the leaf ultrastructure and increase the amount of starch in chloroplasts [20]–[23], which may affect photosynthetic capacity.

Plant species differ greatly in response to elevated [CO_2_]. The present research is the first study to report the effect of [CO_2_] on leaf photosynthetic physiology, chlorophyll fluorescence and mesophyll cells ultrastructure in *I. indigotica* under open-air conditions. This study aims to address the following questions: (1) Will the leaf photosynthetic physiology, chlorophyll fluorescence and leaf mesophyll cell ultrastructure of *I. indigotica* be altered under elevated [CO_2_] and is there a correlation between them? (2) Will elevated [CO_2_] improve photosynthetic ability of *I. indigotica* and what is its implications for the yield and the functional ingredient adenosine of indigowoad root and leaf?

## Results

### *P*_N_ and Gas Exchange Parameters

When photosynthesis was measured at the respective growth [CO_2_], *P*_N_ of upper most fully-expended leaves for *I. indigotica* was increased by 13.1, 22.8 and 27.1% under elevated [CO_2_] 36, 53 and 84 days after sowing, respectively; whereas g_s_ and *T*r were not affected by elevated [CO_2_](Table 1). Elevated [CO_2_] increased WUE by 1.3, 28.9 and 20.7%, at 36, 53 and 84 days after sowing, respectively. *J*_max_ of leaves under elevated [CO_2_] were significantly higher than ambient [CO_2_] by 2.2, 7.3 and 20.2%, respectively, but *V*_c,max_ did not change (Table 1). The interactive effect between [CO_2_] and growth stage on *P*_N_, g_s_, *T*r, WUE, *V*_c,max_, *J*_max_ was not significant (Table 1).

**Table 1:** Effects of elevated [CO_2_] on gas exchange parameters in the last fully-expanded leaves of *I. indigotica.*

Days after sowing(d)	Growth [CO_2_]	*P*_N_ [µmol(CO_2_) m^–2 ^s^–1^]	g_s_ [mmol(H_2_O)m^–2 ^s^–1^]	Tr [mmol m^–2 ^s^–1^]	WUE [µmol mmol^–1^]	V_cmax_ [µmolm^–2 ^s^–1^]	J_max_ [µmolm^–2 ^s^–1^]
36	ambient	18.49±0.57	1.22±0.09	8.42±0.44	2.30±0.06	70.69±2.80	99.2±5.48
	FACE	20.92±0.34	1.38±0.16	9.02±0.28	2.33±0.11	72.35±3.98	101.43±2.74
53	ambient	19.08±0.91	1.25±0.05	9.50±0.19	2.01±0.06	85.03±5.53	115.31±5.22
	FACE	23.43±1.15	1.12±0.05	9.05±0.32	2.59±0.12	81.07±6.44	123.72±11.36
84	ambient	16.88±0.64	0.32±0.09	3.79±0.55	4.59±0.51	85.67±10.07	139.21±9.02
	FACE	19.76±0.83	0.26±0.03	3.61±0.34	5.54±0.42	92.60±3.81	167.34±5.20
*P* values	Growth stage	0.01	0.00	0.00	0.00	0.03	0.00
	CO_2_	0.00	0.94	0.96	0.04	0.76	0.05
	Growth stage*CO_2_	0.46	0.29	0.37	0.30	0.67	0.20

Measurement was taken on their respective [CO_2_]. Values are means ± standard error of variables across the three replicates; three plants were tested in each plot. The statistical significance level for the effects of [CO_2_] treatment, growth stage and their interaction was tested. *P*_N_ - net photosynthetic rate; g_s_- stomatal conductance; *T*r - transpiration ratio; WUE- water use efficiency; *V*_c,max_- maximum velocity of carboxylation; *J*_max_ - maximum rate of electron transport.

### Chlorophyll Fluorescence

Maximum quantum efficiency (*Fv/Fm*) ranged from 0.82 to 0.84 and was not affected by elevated [CO_2_]. Elevated [CO_2_] significantly increased intrinsic efficiency of PSII (*Fv’/Fm’*) and quantum yield of PSII (*ΦPSII*) by 5.3 and 10.2%, respectively. Elevated [CO_2_] decreased leaf non-photochemical quenching (*NPQ*) by 14.7%, but did not affect the proportion of open PSII reaction centers (*qP*) in leaves (Table 2).

**Table 2:** Effects of elevated [CO_2_] on chlorophyll fluorescence parameters in the last fully-expanded leaves of *I. indigotica.*

Days after Sowing(d)	Growth [CO_2_]	Fv/Fm	Fv’/Fm’	ΦPSII	qP	NPQ
36	ambient	0.84±0.00	0.53±0.01	0.31±0.00	0.59±0.02	1.89±0.07
	FACE	0.84±0.00	0.57±0.01	0.33±0.01	0.59±0.02	1.43±0.13
53	ambient	0.82±0.00	0.49±0.02	0.28±0.02	0.57±0.01	1.52±0.08
	FACE	0.82±0.01	0.53±0.01	0.33±0.01	0.61±0.02	1.33±0.05
84	ambient	0.82±0.01	0.49±0.01	0.29±0.01	0.59±0.03	2.39±0.14
	FACE	0.82±0.01	0.49±0.02	0.31±0.02	0.62±0.02	2.19±0.22
*P* values	growth stage	0.04	0.00	0.26	0.52	0.00
	CO_2_	0.95	0.02	0.02	0.10	0.02
	Growth stage*CO_2_	0.83	0.35	0.54	0.39	0.51

Values are means ± standard error of variables across the three replicates; six plants were taken in each plot. The statistical significance level for the effects of [CO_2_] treatment, growth stage and their interaction was tested. *Fv/Fm*- maximum quantum efficiency of PSII; *Fv’/Fm’*- intrinsic efficiency of PSII; *ΦPSII*- quantum yield of PSII; *NPQ*- non-photochemical quenching; *qp*- proportion of open PSII reaction centers.

### Leaf Mesophyll Cells

**Figure 1:**
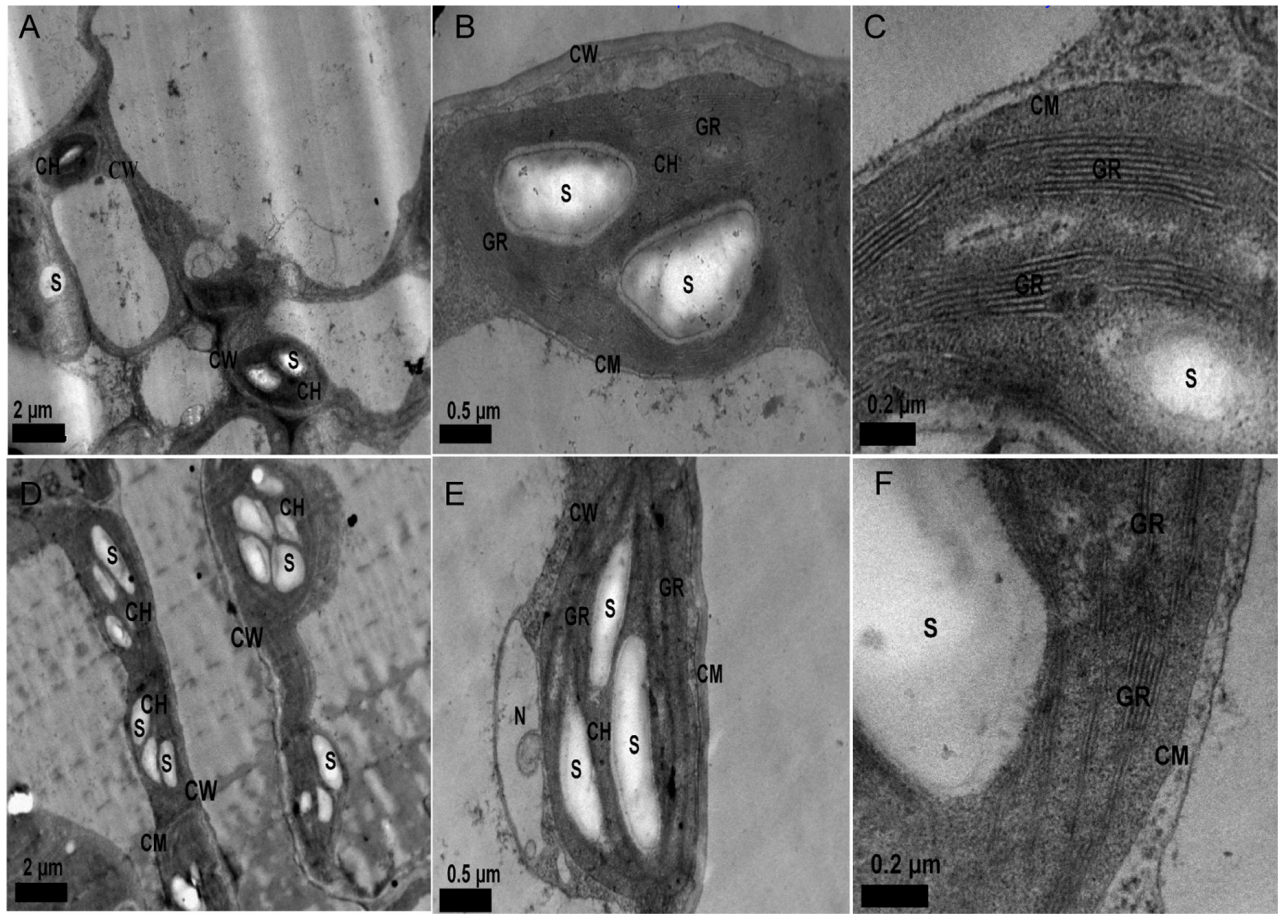
Effects of elevated [CO_2_] on chloroplast ultrastructure in mesophyll cells of *I. indigotica* leaf. *A*–*C*: Chloroplast ultrastructure of *I. indigotica* leaf grown in ambient (×8,000, ×30,000, ×80,000). *D*-*F*: Chloroplast ultrastructure of *I. indigotica* leaf grown under elevated [CO_2_] (×8,000, ×30,000, ×80,000). S: starch grain; GR: grana layer; CM: chloroplast membrane; CH: chloroplast; CW: cell wall; N: nucleus.

Elevated [CO_2_] increased the number of starch grains per chloroplast profile and the area per starch grain by 150.0% and 144.3%, respectively (Table 3 and Fig.1 *A*, *B*, *D*, *E*). The structural membrane of the chloroplast was not affected by elevated [CO_2_]. Similarly, the structural membranes of grana layer and stroma thylakoid were intact, but much tighter under elevated [CO_2_] than those under ambient [CO_2_] (Fig. 1 *B*, *C*, *E*, *F*).

**Table 3:** Effects of elevated [CO_2_] on chloroplast feature of *I. indigotica.*

Growth [CO_2_]	Number of starch grains per chloroplast profile	Area per starch grain (µm^2^)
ambient	1.5±0.29	0.74±0.08
FACE	3.75±0.48	1.81±0.12
increase	150.0%	144.3%
*P* value	0.01	0.00

Values are means ± standard error from three plants. Number of starch grains per chloroplast profile was determined from 50 chloroplasts. Area per starch grain was determined from 50 starch grains. The statistical significance level for the effects of [CO_2_] treatment was tested.

### Biomass and the Content of Adenosine in Root

**Figure 2:**
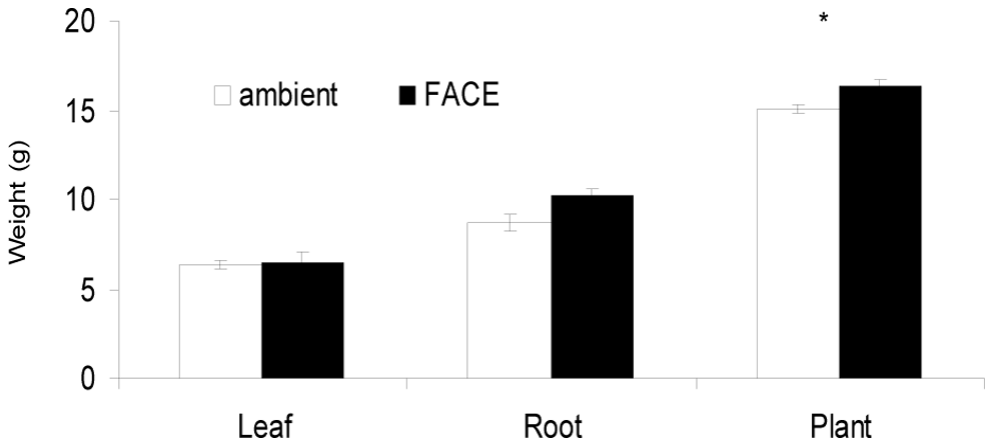
Effects of elevated [CO_2_] on dry weight of leaf and root per plant. Each bar represents the standard error of the difference between treatments (*n* = 3). *p≤0.05.

**Figure 3:**
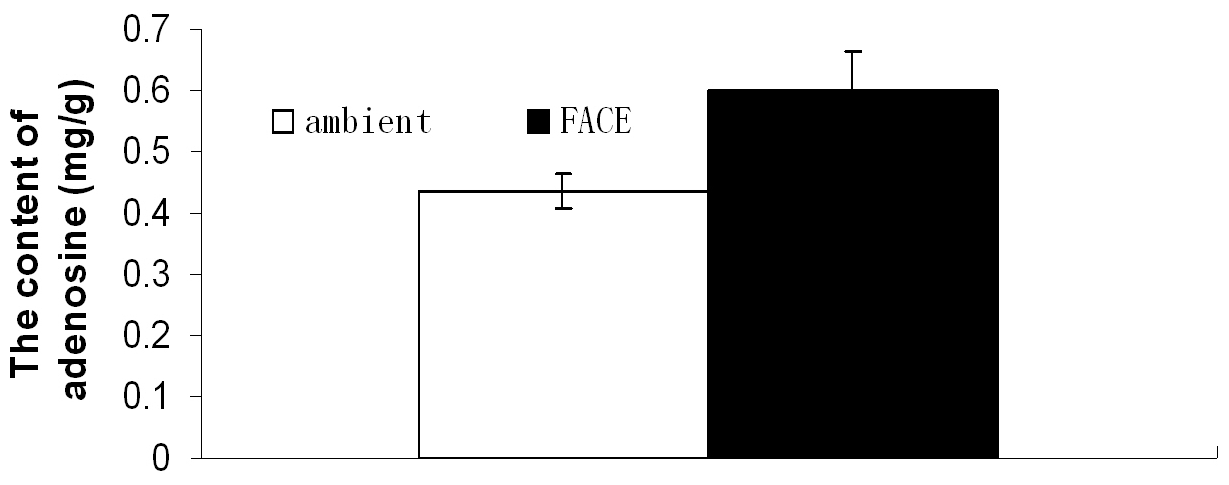
Effects of elevated [CO_2_] on the content of adenosine in root. Each bar represents the standard error of the difference between treatments (*n* = 3).

Elevated [CO_2_] increased the weight of root per plant by 17.4% (*P* = 0.06), but had no significant effect on the weight of leaves. The mean weight per plant was 15.1 g/plant and 16.4 g/plant under ambient [CO_2_] and elevated [CO_2_], respectively. The total weight per plant was significantly increased by 8.8% (*P*<0.05) (Fig.2). The mean adenosine contents in root were 0.44 mg/g and 0.59 mg/g under ambient [CO_2_] and elevated [CO_2_], respectively, and was not significantly affected by elevated [CO_2_] (Fig.3).

## Discussion and Conclusion

The enhancement of photosysnthesis by elevated [CO_2_] diminishes over time (days to years), termed photosynthetic acclimation [4]. Photosynthetic acclimation has been well documented in C_3_ plants *e.g.* rice [24], [25], soybean [23], [26] and wheat [27], and the response varied with plant species, cultivars, developmental stages and environmental conditions [2], [23]. Quantum yield of PSII(*ΦPSII)* may serve as a quantitative indicator of electron transport through PSII, which is related to the photochemical efficiency of PSII [28]. Non-photochemical quenching (*NPQ*) indicates plants dissipate energy by the means of thermal energy rather than linear electron transport. Non-photochemical quenching increases dramatically when sink is limited (no development of new sinks) [29]. In our study, increases in P_N_ and *J*_max_ under elevated [CO_2_] coincided with increases in *ΦPSII*, the efficiency of light harvesting and energy transduction of open *PSII* centres (*Fv’/Fm’*). *V*_c,max_, the maximum quantum efficiency of PS*II*(*Fv/Fm)* and *qP* (an estimate of open *PSII centres*) showed no significant change under elevated [CO_2_], but *NPQ* was decreased. These results are partially in agreement with the study by Tausz-Posch et al [28], which showed increases in *ΦPSII, Fv’/Fm’* and *qP* of wheat grown under elevated [CO_2_] and mild or moderate stress conditions. During the first year of cultivation, *I. indigotica* was only in its vegetative growth stage, plant growth might require large amount of photosynthates, hence no limitation in sink. Therefor *NPQ* was decreased and more energy was used in linear electron transport (*ΦPSII)* under elevated [CO_2_].

The increase in the number and size of starch grain in chloroplast indicated that more photosynthates were produced through increased photosynthesis under elevated [CO_2_]. More photosynthates would restrain photosynthesis if no new sink is available to accommodate superfluous assimilation [23], [30], [31], resulting in photosynthetic acclimation. Photosynthetic acclimation occurs in rice flag leaves under FACE because N content in the leaves was reduced under a high N demand for reproductive development, but not in the eighth leaf stage when vegetative growth occured [32]. Photosynthetic acclimation occurred at the seedling stage (R5) in soybean cultivar Zhonghuang 13, but not in the other soybean cultivar Zhonghuang 35. Acclimation did not occur for Zhonghuang 35, because extra C sink was developed and the photosynthesis was not restricted under elevated [CO_2_] [23]. Acclimation to high [CO_2_] was not apparent when the ratio of sink to source activity was increased, but it was observed when the ratio was reduced [30]. Photosynthetic acclimation did not occur in *I. indigotica* under elevated [CO_2_] in the first year when new C sinks developed.

The [CO_2_]-induced increased photosynthesis increased biomass by 8.8% in our experiment. Similar [CO_2_] effects were also observed for other species of the *Brassicaceae* family such as *Arabidopsis thaliana* and Chinese cabbage [17], [18]. Compared to ambient [CO_2_], elevated [CO_2_] significantly increased the photosynthetic rate of *Arabidopsis thaliana* by an average of 17.1% for three generations, resulting in increased reproductive mass, total mass per plant, and percentage of reproductive mass per plant [14]. The *P*_N_ of Chinese cabbage was increased by 39% under elevated [CO_2_], and the dry weight per plant by 28.2% [15]. The content of adenosine, one of the main medical ingredients of indigowoad root, was not affected by elevated [CO_2_], though the total yield of adenosine per plant increased by 58.3% (p<0.05) (data not shown) due to the increase in the yield of the root under elevated [CO_2_].

Climate, soil type and fertilizer application influence the growth of herbal medicine plant, and its active ingredients [33]. Global temperature may increase with [CO_2_] in future, which could lead to climate change with perturbations like more severe and frequent drought events [28]. [O_3_] could also increase with the development of industries. Plant growth and metabolism could be affected by combined factors including elevated [CO_2_], increased temperature, drought and elevated [O_3_]. Any further increase in night temperatures, or events of high temperatures decreased rice yields drastically [34], [35]. Increased [CO_2_] does not compensate for the negative effects on yield of higher temperature and [O_3_] in oilseed rape [36]. Increased temperature, drought and elevated [O_3_] would reduce the fertilization effect of elevated [CO_2_] [37], [38]. The effect of growth, metabolism, effective compositions of *I. indigotica* with climate change including elevated [CO_2_], increased temperature, drought and elevated [O_3_] would be a new area to explore in future research.

The decrease in *g*_s_ under elevated [CO_2_] is associated with increased water use efficiency [39]. There was no significant reduction in *g*_s_ of *I. indigotica* under elevated [CO_2_], but the water use efficiency increased by 17.5% because of the increase in *P_N_*. These indicate that the herbal plant may be tolerant to some extent of the drought conditions potentially accompanied with elevated [CO_2_].

In addition, the structure of grana layer and stroma thylakoid membranes were intact, but much tighter than those under ambient [CO_2_]. A correlation might exist between the conformation of stroma thylakoids and starch grain accumulation, because the chloroplast was occupied by an excess of large starch grains [21]. The grana layer and the membrane structure under elevated [CO_2_] were not intact in the leaf, which contributed to photosynthetic acclimation for soybean [23]. *P*_N_, *J*_max_, *Fv’/Fm’* and *ΦPSII* under elevated [CO_2_] were improved, and photosynthetic acclimation was not encountered for *I. indigotica*,which is in accordance with the intact structural of grana layer and stroma thylakoid membranes.

In summary, *P*_N_, WUE, *J*_max_, *Fv’/Fm’* and *ΦPSII* of upper most fully-expanded leaves in *I. indigotica* were increased under elevated [CO_2_]. We concluded that photosynthetic ability of *I. indigotica* was improved through the change of leaf photosynthetic physiology and chlorophyll fluorescence under elevated [CO_2_], which led to the increased in the yield of indigowoad root. The content of adenosine in indigowoad root was not altered under elevated [CO_2_].

## Materials and Methods

### Site Description

The study was conducted at the Mini-FACE facility developed by IEDA (Institute of Environment and Sustainable Development in Agriculture) located at an experimental station of the Chinese Academy of Agricultural Sciences at Changping, Beijing, China (40.13°N, 116.14°E). The operational procedures of the facility were as described in a previous experiment [23]. The long term average rainfall and temperature during the *I. indigotica* growing season were 475 mm and 21.3°C, respectively.

### *I. indigotica* Cultivation and Irrigation

*I. indigotica* were sown on 26 June 2011 in 40 cm pots (25 cm depth). Three plots were with ambient [CO_2_] of 411±15 µmol mol^–1^, and another three plots with elevated [CO_2_] of 550±19 µmol mol^–1^, simulating the expected environment in 2050. Three plants were grown in each pot and four pots were included in every plot. The soil was a clay loam with a pH (1∶5 soil:water) of 8.6 and contained 1.21% organic carbon (C) and 0.11% total N. Fertilizers were applied at sowing at the rates of 3.68 g N per plot and 4.08 g P per pot. Irrigation equivalent to 10–20 mm of rainfall was applied every 2–3 days after the seedling emerged from soil.

### Transmission Electron Microscopy

For anatomical and ultra-structural investigations, samples from the upper most fully-expanded leaves facing the sunlight were collected (three plants per plot) on 19 August 2011 (between 10∶00 and 12∶00, at 11-leaf stage). Leaves were observed using an electron microscope (JEOL JEM-2100F) operating at 80 kV as described previously [23].

### Gas Exchange Measurements

Measurements of *P*_N_ (Net photosynthetic rate) vs C_i_ (intercellular CO_2_ concentration) were conducted 36, 53 and 84 days after sowing. One of the upper most fully-expanded leaves was randomly selected. The number of leaves on plants was 7, 11 and 18 correspondingly. Gas exchange measurements were conducted using portable gas exchange systems (LI-COR 6400; LI-COR, Lincoln, Neb, USA). The [CO_2_] in the leaf chamber was controlled by the LI-COR CO_2_ injection system, and an irradiance of 1,400 µmol photons m^–2^s^–1^ was supplied using an built-in LED lamp (red/blue). Temperature in the 2×3×2.5 cm^3^ leaf chamber was set at 25°C, and the actual temperature ranged from 25 to 28°C. The vapour pressure deficit (VPD) on the leaf surface was between 1.9 and 2.1 kPa. The [CO_2_] surrounding the leaf for all control and treatment leaves was controlled across the series of 550, 400, 300, 200,100, 50, 400, 550, 600, 700, 800, 1000, 1200 and 550 µmol mol^–1^, and measurements were recorded after equilibrium was reached. Measurements were made between 9∶00 and 14∶00 local time. Each individual curve took approximately 35 min to complete. Values for *P*_N_ and C_i_ were used to calculate *V*_c,max_ (Maximum velocity of carboxylation) and *J*_max_ (Maximum rate of electron transport) values using the model and software provided by Sharkey et al [40]. *P*_N_, *T*r (transpiration ratio), WUE (water use efficiency, WUE = *P*_N_/*T*r) and *g*_s_ (stomatal conductance) were also measured at the same irradiance, temperature and vapour pressure when the measurements of *P*_N_ vs C_i_ were conducted. [CO_2_] in the leaf chamber was set to 400 µmol mol^–1^ for current [CO_2_] treatment and 550 µmol mol^–1^ for elevated [CO_2_] treatment, and one upper most fully-expanded leaf was measured per pot.

### Chlorophyll fluorescence

The photosynthetic performance of upper most fully-expanded leaves was assessed in terms of the chlorophyll a fluorescence parameter *Fv/Fm* (Maximum quantum efficiency of PSII), *Fv’/Fm’* (Intrinsic efficiency of PSII), *ΦPSII* (Quantum yield of PSII), *qP* (Proportion of open PSII reaction centers ), *NPQ* (Non-photochemical quenching) using a miniaturized pulse-amplitude modulated fluorescence analyzer (Mini-PAM, Walz, Effeltrich, Germany) with a leaf clip holder as described by Bilger et al [41]. The fluorescence parameter was measured at incident PPFD between 9∶00 and 15∶00 h over 3 consecutive days, for a total of 6 individual leaves in each plot. F0′ and Fm′ of darkness-adapted leaves were measured between 23∶00 and 01∶00 h on the same day. The high light flash used to measure saturated fluorescence had a PPFD (Photosynthetic Photon Flux Density) of 4,000 µmol m^−2^s^−1^and a duration of 800 ms. All chlorophyll fluorescence parameters were calculated as described by Rascher et al [42].

### Harvesting

*I. indigotica* plants were harvested on 8 October 2011 (102 days after sowing). All the plants were separated into above ground parts and roots, air dried and weighed.

### The Content of Adenosine

**Figure 4:**
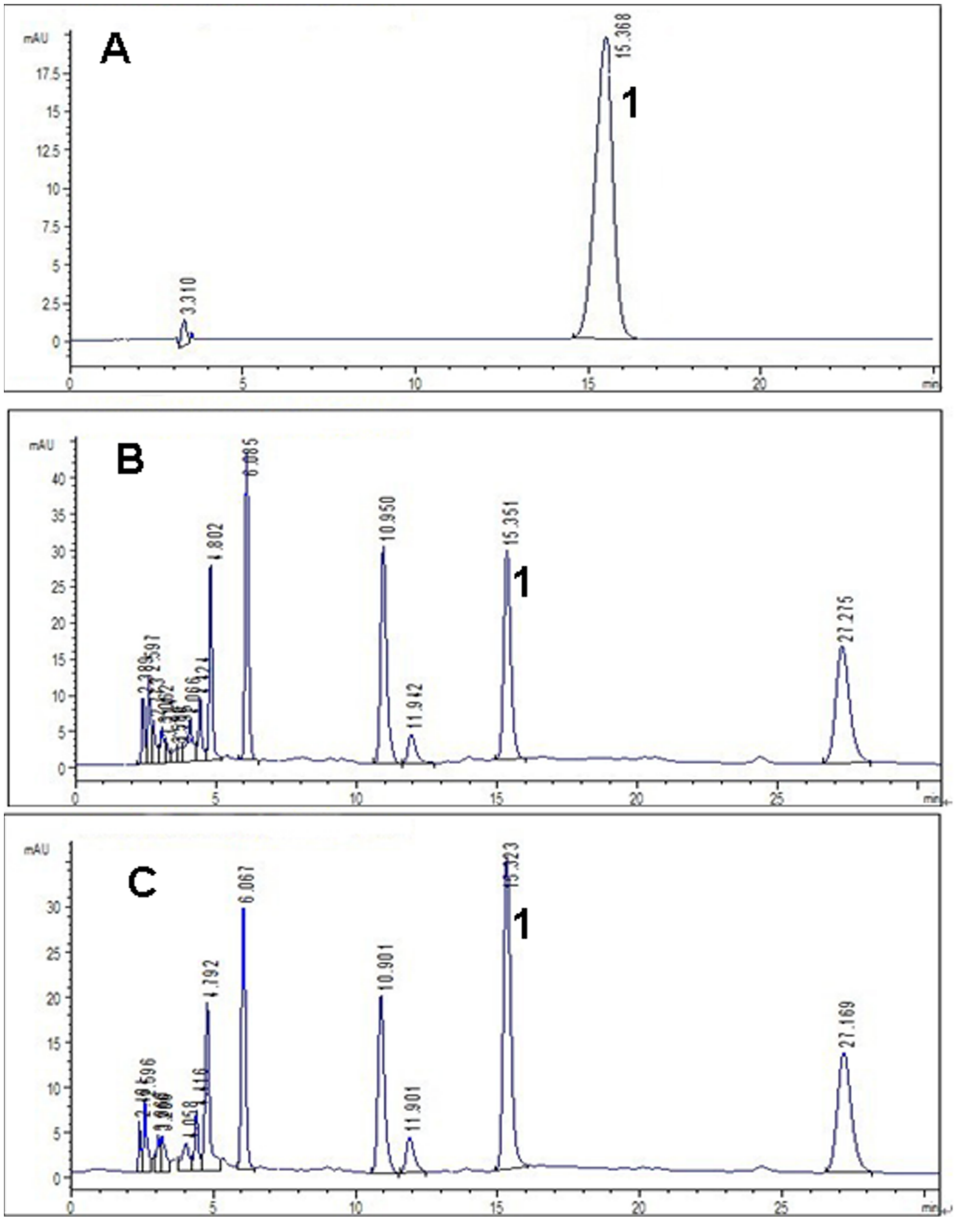
HPLC chromatograms of adenosine standard, samples of the root under ambient [CO_2_] and elevated [CO_2_]. Peak #1 is adenosine commercial standard. A: Representative chromatogram for adenosine standard (The contents of adenosine were 0.0272 mg/ml. Five concentrations of adenosine standard have been measured for the standard curve: 0.0068, 0.0136, 0.0272, 0.0544 and 0.068 mg/ml). B: Root sample chromatogram under ambient [CO_2_](one out of three replicates). C: Root sample chromatogram under elevated [CO_2_] (one out of three replicates).

Standard of adenosine were purchased from National institutes for food and drug control (Beijing, China). All the roots were dried at 50°C until constant weight is reached. Samples were pulverized to 80 meshes. 1.0 g of pulverized powder was ultrasonically extracted with 25 ml of methanol for 30 minutes. The supernatant was filtered and cooled to ambient temperature. The obtained solution extracted from the root and standards solution were filtered through a syringe filter (0.45 µm) and aliquots (10 µl) were subjected to HPLC analysis by Agilent 1100 series HPLC-DAD system (Agilent, Palo Alto, CA, USA). Agilent Zorbax Eclipse XDB-C_18_ column (4.6×250 mm, 5 µm, USA) was used. The mobile phase was a mixture of methanol/water (8∶92, v/v). Elution was performed at a solvent flow rate of 1 ml/min. Detection was performed with a variable-wavelength UV detector (L-4250) at 260 nm [13]. HPLC chromatograms of adenosine standard and root samples under ambient [CO_2_] and elevated [CO_2_] were shown in Fig 4.

### Statistical Analysis

All experimental data presented were analyzed by analysis of variance at 0.05 probability level using SAS System 8.1.
